# Dataset on multiregional variations of Bangla language (BD-Dialect)

**DOI:** 10.1016/j.dib.2026.112654

**Published:** 2026-03-03

**Authors:** Anika Rahman, Nafesha Hasan Muna, Masuma Saba Prity

**Affiliations:** Department of Computer Science and Engineering, Stamford University Bangladesh, Dhaka 1217, Bangladesh

**Keywords:** Bangla dialects, Linguistic dataset, Regional language variation, Dataset validation, Natural language processing, Multilingual corpus, Dialectal diversity

## Abstract

The **BD-Dialect dataset** presents a comprehensive multiregional linguistic resource for Bangla and its major regional dialects, designed to support research in computational linguistics, dialectology, and natural language processing (NLP). The dataset includes aligned translations across **Standard Bangla** and five major dialects—**Noakhali, Sylhet, Chittagong, Rajshahi,** and **Mymensingh**—alongside **English translations** to facilitate cross-linguistic comparison. Data were collected from two sources: **native speaker interviews** and **regional literature**, ensuring both lexical richness and regional authenticity. The final dataset consists of **two CSV files** (words and clauses), each containing **950 aligned entries** structured under seven columns: Standard Bangla, Noakhali, Sylhet, Chittagong, Rajshahi, Mymensingh, and English Translation. Preprocessing and formatting were conducted using **Python in Google Colab**, followed by validation by **three native speakers per dialect** to ensure linguistic accuracy and consistency. The dataset and preprocessing scripts are **publicly available in Mendeley Data** under DOI: 10.17632/k769s4vk5z.2, providing an open-access resource for developing dialect recognition models, translation systems, and comparative linguistic research in Bangla.

Specifications Table

**Table:** 

Subject	Computer Sciences
Specific subject area	Linguistic dataset creation and dialectal variation in Bangla language
Type of data	Table, Chart, Figure Raw, Cleaned, Processed
Data collection	Data were collected from **native speakers**, and **regional literature** across five major dialectal regions of Bangladesh: Noakhali, Sylhet, Chittagong, Rajshahi, and Mymensingh. Each entry was recorded, transcribed, and manually translated into Standard Bangla and English. The data were then cleaned, standardized, and validated by three native speakers per dialect to ensure linguistic and semantic accuracy.
Data source location	Data collection took place in Bangladesh. Dataset and scripts are stored under the Department of Computer Science and Engineering, Stamford University Bangladesh, Bangladesh.
Data accessibility	Repository name: Mendeley Data Data identification number: 10.17632/k769s4vk5z.2 Direct URL to data: https://data.mendeley.com/datasets/k769s4vk5z/2 Instructions for accessing these data: The dataset is publicly available under open access (CC BY 4.0 license) on Mendeley Data. Users can download both CSV files (words and clauses) along with preprocessing scripts directly.
Related research article	None (the dataset currently stands as a standalone contribution).

## 1 Value of the Data

• Comprehensive Multiregional Coverage:
  This dataset provides parallel linguistic data for **Standard Bangla** and **five major dialects**—Noakhali, Sylhet, Chittagong, Rajshahi, and Mymensingh—making it one of the first publicly available resources capturing such extensive dialectal diversity in the Bangla language [1,2].
• Facilitates NLP Research in Low-Resource Languages:
  The dataset can serve as a foundational resource for **machine translation, dialect identification, speech recognition**, and **text normalization** tasks, supporting researchers working on underrepresented South Asian languages [3,4].
• High Linguistic Reliability:
  Each entry was **validated by three native speakers per dialect**, ensuring strong linguistic consistency and semantic accuracy across translations.
• Supports Cross-Dialectal and Cross-Lingual Analysis:
  By including **English translations**, the dataset enables comparative linguistic research, transfer learning, and multilingual corpus analysis [5].
• Open Access and Reusability:
  The dataset and associated preprocessing scripts are **freely available on Mendeley Data** under a **CC BY 4.0 license** [6], allowing researchers to reuse, modify, or extend the data for educational and research purposes.

## 2 Background

Bangla, one of the most widely spoken languages in South Asia, exhibits substantial dialectal diversity across different regions of Bangladesh [1,7]. Despite this linguistic richness, there remains a lack of comprehensive and structured resources representing dialectal variations for computational and linguistic analysis [8,2]. The motivation behind compiling the BD-Dialect: A Multiregional Bangla Language Dataset was to create a foundational resource that captures regional speech and lexical patterns to support research in natural language processing, sociolinguistics, and speech technology development [9,10].

The dataset construction was guided by linguistic theory on regional variation and phonological shifts [7], combined with modern data collection techniques involving native speaker interviews and text sources [11,12]. Methodologically, the dataset integrates standardized transcription and normalization to ensure consistency and usability in machine learning applications.

This dataset extends prior efforts in Bangla language resources [[3], [8]] by systematically documenting dialectal features from multiple regions, enabling comparative studies and dialect-aware NLP model training. It complements ongoing research on Bangla linguistic diversity by offering open access to a curated, annotated, and regionally balanced dataset.

## 3 Data Description

The BD-Dialect: A Multiregional Bangla Language Dataset consists of two primary CSV files — Words.csv and Clauses.csv — each containing 950 rows. Every entry includes parallel translations across one standard Bangla form and five major dialects: Noakhali, Sylheti, Chittagong, Rajshahi, and Mymensingh, along with English translations for cross-linguistic reference.

The BD-Dialect dataset consists of two CSV files — one for words and another for clauses — each containing 950 rows across seven language columns. Table 1 provides a structural overview of the dataset. Each CSV file maintains a consistent schema to facilitate comparison, linguistic analysis, and computational modelling.

**Table 1:** Overview of the BD-Dialect dataset structure and sample entries showing the seven language columns.

SL No.	Language	Description
1	Standard Bangla Language	The standardized Bangla form of the word or clause
2	English Translation	English translation of the respective Standard Bangla entry
3	Noakhali Language	Dialectal form used in Noakhali region
4	Sylheti Language	Dialectal form used in Sylhet region
5	Chittagong Language	Dialectal form used in Chittagong region
6	Rajshahi Language	Dialectal form used in Rajshahi region
7	Mymensingh Language	Dialectal form used in Mymensingh region

To illustrate the dataset's structure and content, Table 2 shows five anonymized sample entries.

**Table 2:** Sample entries from the BD-dialect dataset.

The dataset repository also includes:

• Preprocessing scripts developed in Python (Google Colab), covering text cleaning, normalization, and validation steps.
• Metadata file describing the data collection process, dialect coverage, and native speaker validation summary.
• Workflow diagram illustrating the entire methodology — from data collection through analysis and visualization.

A small set of audio recordings from native speakers were collected and shared to support internal verification of phonetic and pronunciation consistency. All data files are available through Mendeley Data under DOI: 10.17632/k769s4vk5z.2.

## 4 Experimental Design, Materials and Methods

**Fig. 1:**
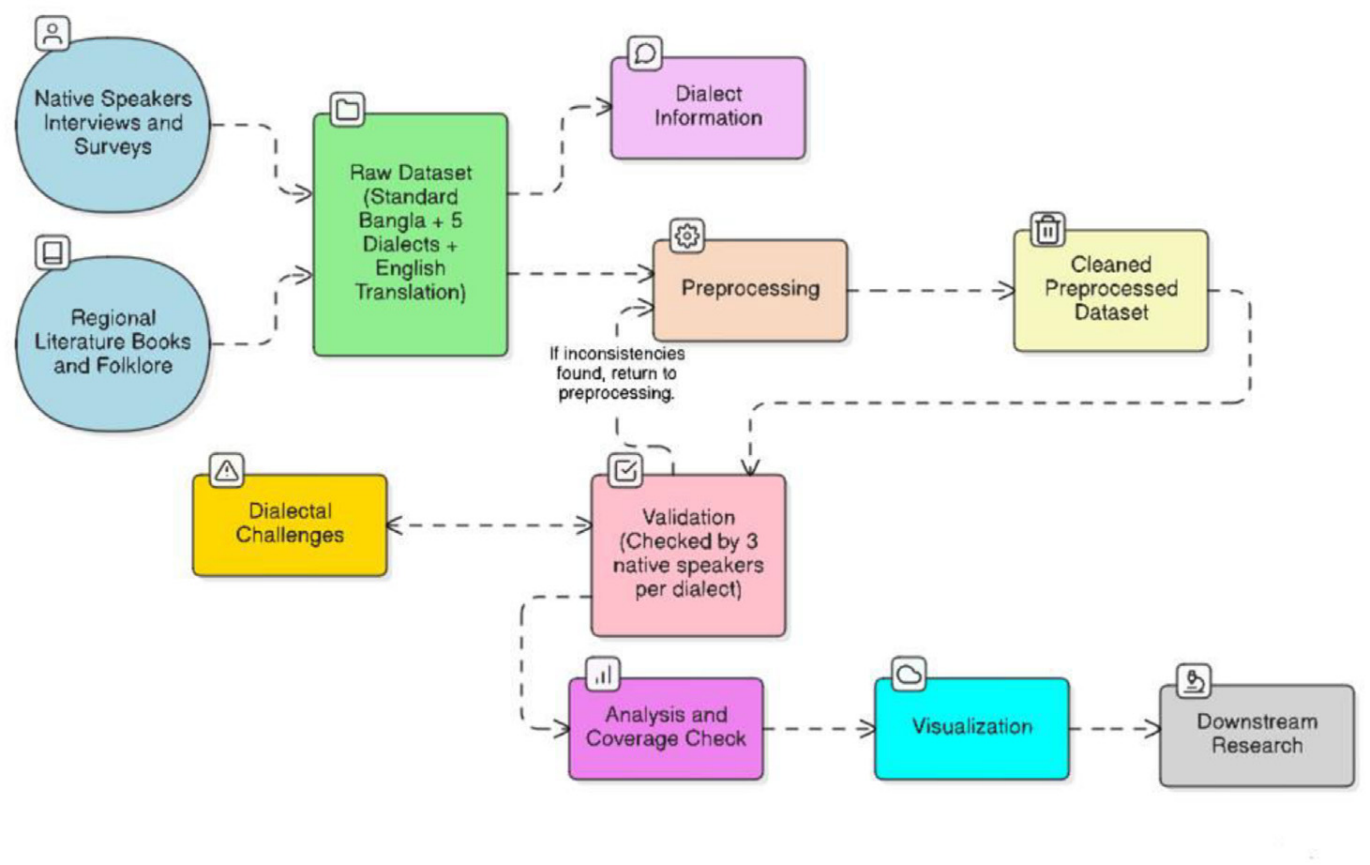
Workflow diagram showing the overall process of data collection, preprocessing, and compilation for BD-Dialect.

The dataset construction followed a multi-stage process, as visualized in the Bangla Dialect Dataset Construction **Workflow diagram**. Fig. 1 summarizes the overall methodology, illustrating the sequential stages from data collection to final repository submission.

### 4.1 Data collection

Two complementary sources were used to ensure dialectal diversity and reliability:

• Native Speakers (Interviews and Surveys): Dialectal variations were elicited from native speakers representing each of the five regions. Responses were recorded, transcribed, and verified. A few pilot audio recordings were collected to confirm phonetic authenticity.
• Regional Literature (Books and Folklore): Authentic words and clauses were identified from classical Bengali literature and folklore. To ensure ethical sourcing and full copyright compliance, only works that are historically recognized as being in the public domain were consulted. These included digitized public-domain texts accessed via the National Digital Library of India (NDLI) and the Internet Archive, where each item was individually verified for copyright status. The selected materials primarily comprised works by authors from the late 19th and early 20th centuries (e.g., Rabindranath Tagore) and curated folk-tale collections. The texts were used solely for linguistic term identification, after which culturally and dialectally appropriate equivalents were obtained and validated through a native-speaker verification process.

### 4.2 Native speaker sampling and demographic summary

Dialectal data were elicited from native speakers who were born and raised in their respective regions and primarily use the dialect in daily life. Participants were recruited through academic networks at Stamford University Bangladesh. A structured elicitation protocol was used, focusing on everyday vocabulary, common phrases, and culturally relevant concepts. The protocol is provided in Supplementary File S2. Table 3 summarizes participant demographics.

**Table 3:** Native speaker participant summary.

Dialect Region	Number of Speakers	Age Range	Gender (M/F)	Primary Role
Noakhali	3	22–30	3 / 0	Data Elicitation & Validation
Sylhet	3	20–30	0 / 3	Data Elicitation & Validation
Chittagong	3	21–32	0 / 3	Data Elicitation & Validation
Rajshahi	5	21–28	3 / 2	Data Elicitation & Validation
Mymensingh	5	23–30	2 / 3	Data Elicitation & Validation

### 4.3 Raw dataset preparation

All collected entries were compiled into a raw dataset containing Standard Bangla, its dialectal equivalents, and English translations. Initial compilation ensured coverage of both formal and informal registers.

### 4.4 Data preprocessing

Data cleaning was performed using Python scripts in Google Colab, including:

• Removal of duplicates and null values
• Correction of typographical inconsistencies
• Unicode normalization for Bangla scripts
• Consistent formatting across dialect columns

### 4.5 Validation

Each entry was independently validated by three native speakers per dialect who were not involved in its initial elicitation. Validators assessed correctness, naturalness, and context.

Adjudication Protocol: Entries with unanimous approval were accepted directly. In cases of disagreement, validators discussed to reach a consensus. Entries where consensus could not be reached were excluded from the final dataset. Over 95 % of entries were validated unanimously in the first round. This multi-validator, consensus-based protocol ensures the dataset's high linguistic reliability.

### 4.6 Analysis and visualization

Basic analyses were conducted to examine:

• Lexical overlap among dialects
• Unique word and clause counts
• Distribution of word and clause lengths

Visualizations such as word clouds and coverage statistics were generated using Python visualization libraries to provide insight into dialectal richness.

**Fig. 2:**
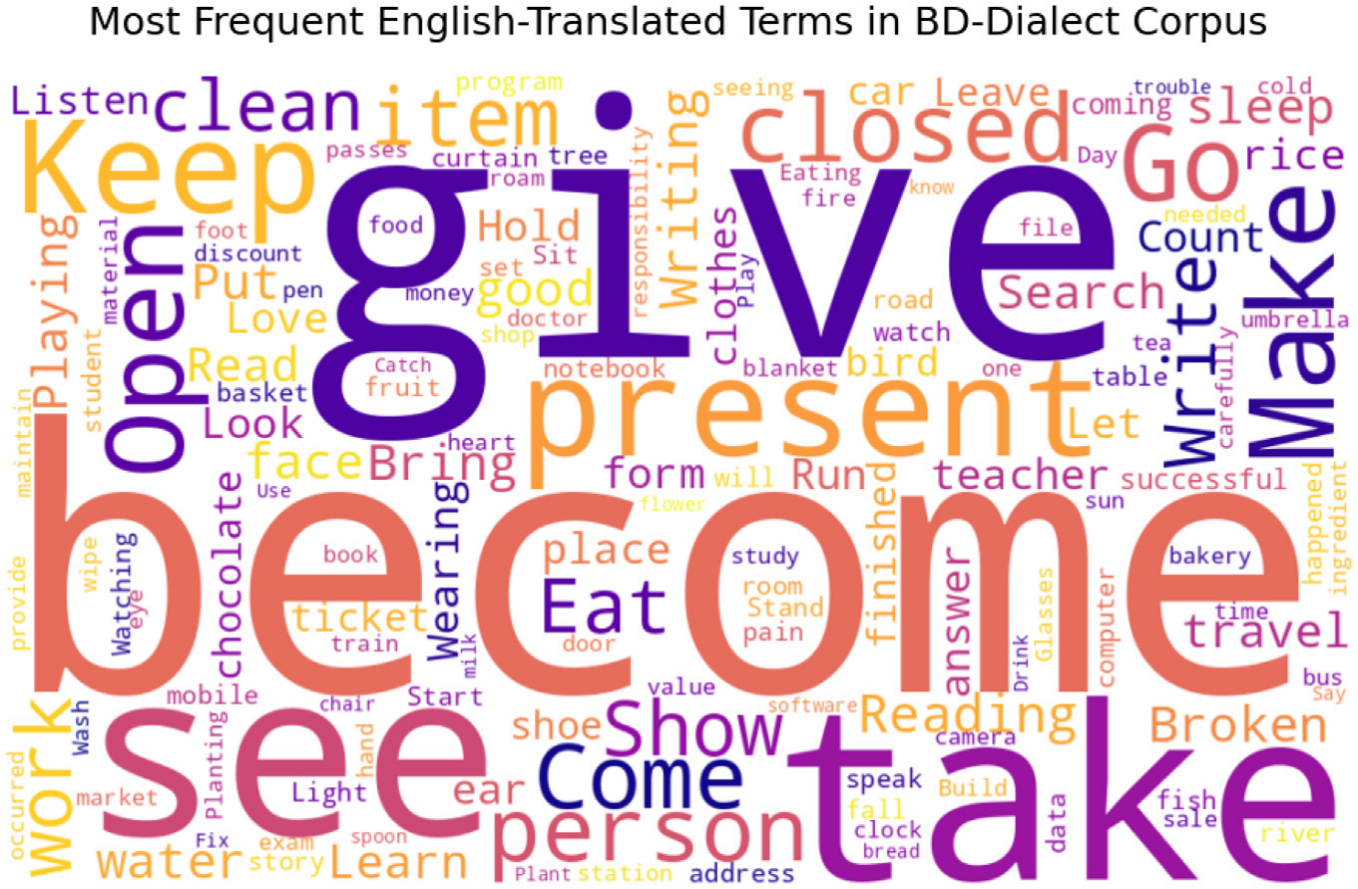
Word cloud of the most frequent English-translated terms in the BD-Dialect corpus.

**Fig. 3:**
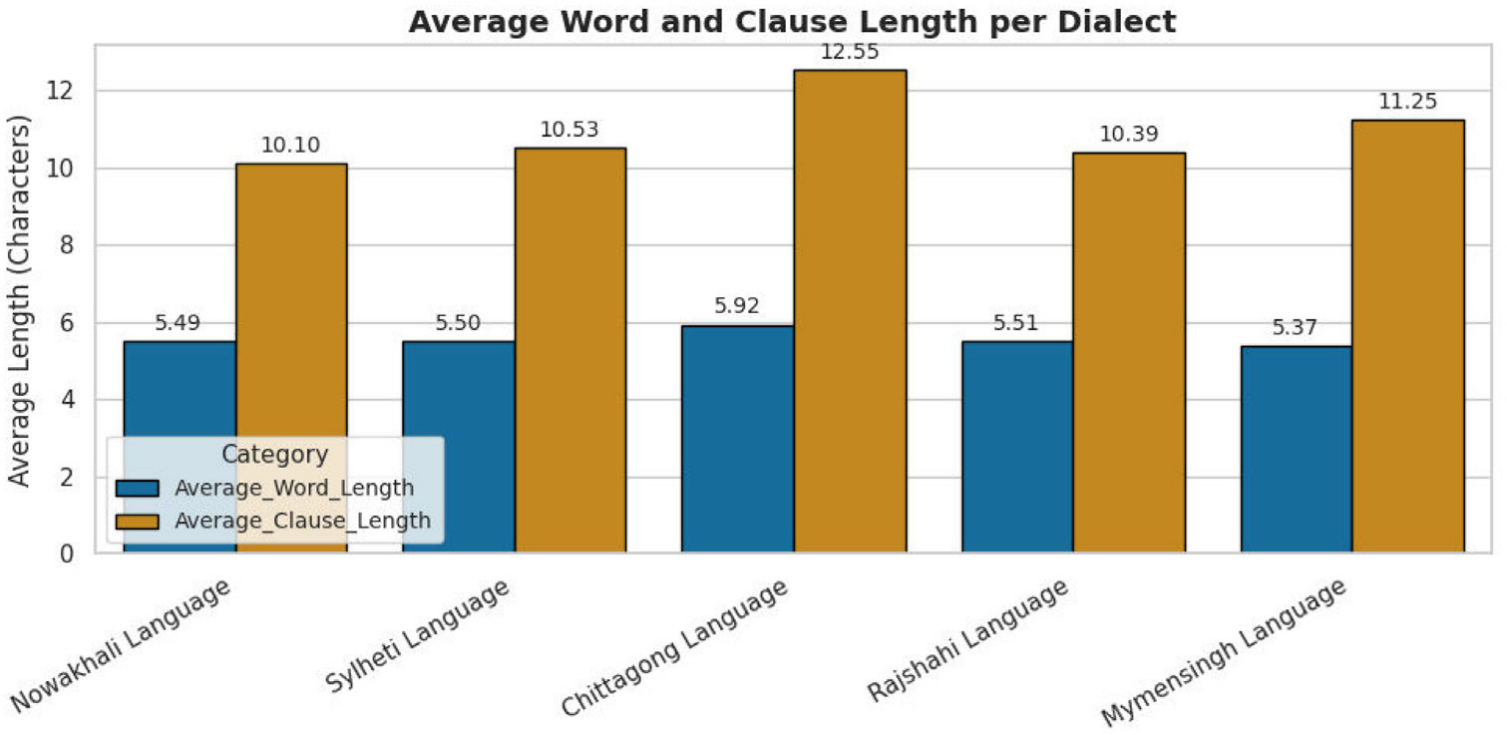
Bar chart showing average word and clause length variation among five dialects.

**Fig. 4:**
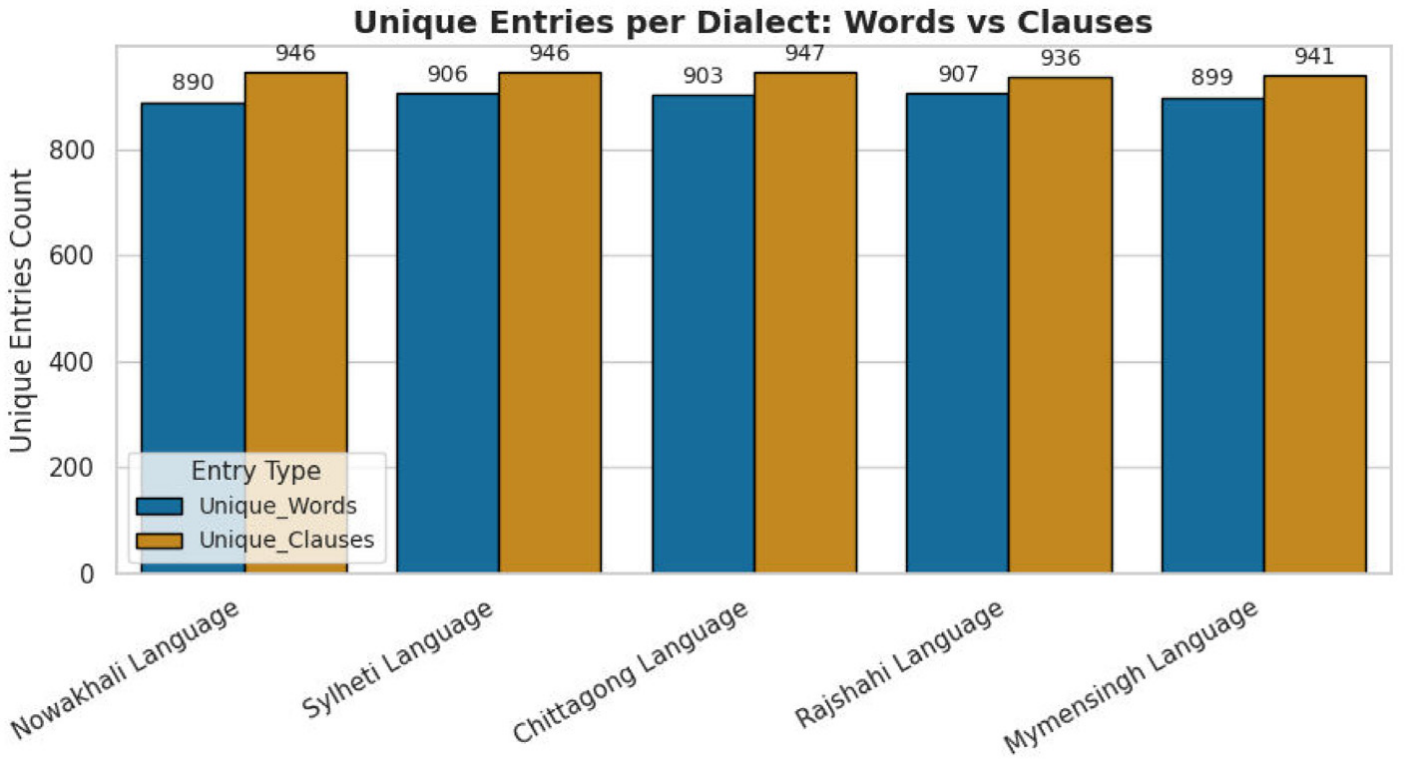
Visualization of unique word and clause counts per dialect indicating lexical diversity.

To visualize the lexical coverage and thematic balance of the corpus, Fig. 2 presents a word cloud of the most frequent English-translated terms. Figs. 3 and 4 further summarize the lexical characteristics, including average word length and unique word count per dialect, providing an overview of dataset coverage and linguistic variability.

**Software Specifications:** All data preprocessing, analysis, and visualization were performed using Python 3.12.12 with the following libraries: pandas (v2.2.2), numpy (v2.0.2), matplotlib (v3.10.0), seaborn (v0.13.2), and wordcloud (v1.9.6).

### 4.7 Documentation and repository submission

All cleaned datasets, preprocessing codes, and documentation were packaged and uploaded to Mendeley Data, ensuring reproducibility and public accessibility.

## Limitations

While the dataset provides broad dialectal coverage across five major Bangla dialects, several limitations exist:

• Partial data collection: Only a subset of dialectal entries was confirmed through direct speaker elicitation; the remainder was sourced from literature, which may include stylistic variation.
• Limited audio representation: A small number of pilot recordings were made for internal validation, and audio data are included in the current public release. A small set of pilot audio recordings from native speakers is included in the repository (in the BD-Dialect_Audio_Samples.zip folder). These were collected for phonetic verification and represent a limited sample, not a comprehensive audio dataset aligned with all 950 text entries.
• Regional imbalance: Some dialects (e.g., Sylheti and Chittagong) have comparatively richer lexical entries due to greater source availability.
• Dialectal nuances: Sub-dialectal variations (micro-regional or sociolectal) are not yet captured but may be included in a future extension of this dataset.

Despite these limitations, BD-Dialect establishes a foundational multilingual and multidialectal resource for Bangla language processing and linguistic studies.

## Ethics Statement

The authors confirm that this study complies with the ethical guidelines outlined in Data in Brief’s Guide for Authors. The dataset compilation primarily involved the collection of publicly available linguistic materials from regional literature, and folklore.

For the limited voluntary contributions from native speakers, informed verbal consent was obtained prior to participation following the ethical protocol documented in the 'Informed_Consent_BD-Dialect.pdf' file. The consent procedure is also summarized in Supplementary File S2. Participants were informed about the non-commercial, academic purpose of the data collection and that their anonymized linguistic contributions would be part of a public dataset. No personally identifiable information (PII) was recorded or stored.

Ethical Approval Statement: According to the policies of Stamford University Bangladesh and the national guidelines for non-invasive linguistic and survey-based research that does not involve sensitive personal data, medical intervention, or vulnerable populations, formal approval from an Institutional Review Board (IRB) or ethics committee was not required for this study. The research involved minimal risk, and all procedures were conducted in accordance with the principles of voluntary participation and data anonymization. Furthermore, to comply with intellectual property guidelines, the dataset compilation excluded copyrighted material from modern news media and online platforms. No copyrighted text is redistributed in the dataset; only derived linguistic annotations and normalized lexical forms are provided.

## CRediT Author Statement

**Anika Rahman:** Conceptualization, Methodology, Data Curation, Visualization, Writing - Original Draft, Visualization, Project Administration, Supervision; **Nafesha Hasan Muna:** Investigation, Data Collection, Data Curation, Validation, Writing - Review & Editing; **Masuma Saba Prity:** Investigation, Data Collection, Data Curation, Validation, Writing - Review & Editing.

## Declaration of generative AI and AI-assisted technologies

During the preparation of this work, the author used ChatGPT (OpenAI) to assist with structuring the research paper, drafting and refining text for clarity. After using this tool, the author reviewed, edited, and verified all content critically. The author takes full responsibility for the content of the published article.

## Data Availability

Mendeley DataBD-Dialect: A Multiregional Bangla Language Dataset (Original data).

## Appendix Supplementary materials

Image, application 1

Image, application 2

## References

[1] Sultana S. (2023). Sociolinguistics research in Bangladesh: prologue and progress. Language in Society in Bangladesh and Beyond.

[2] Sen O. (2022). Bangla natural language processing: a comprehensive analysis of classical, machine learning, and deep learning-based methods. IEEE Access.

[3] Dawn D., Debapratim, Shaikh S.H, Pal R.K (2020). A comprehensive review of bengali word sense disambiguation. Artif. Intell. Rev..

[4] Upama P.B (2024). Proc. 2024 IEEE 48th Annu. Comput. Softw. Appl. Conf. (COMPSAC).

[5] Tareq M. (2023). Data-augmentation for bangla-english code-mixed sentiment analysis: enhancing cross linguistic contextual understanding. IEEE Access.

[6] A. Rahman; H. Muna, Nafesha; M.S Prity (2026), “BD-dialect: a multiregional Bangla language dataset”, Mendeley Data, V2, doi: 10.17632/k769s4vk5z.2. 10.1016/j.dib.2026.112654 PMC12996979 41858330

[7] Karmaker P.R (2019). Dialectical and linguistic variations of Bangla sounds: phonemic analysis. Jagannath Univ. J. Arts.

[8] Chowdhury S. (2025). ChatgaiyyaAlap: a dataset for conversion from chittagonian dialect to standard Bangla. Data Brief.

[9] Hasan M.dN., Azim R., Sharmin S. (2024). Proc. 2024 IEEE Int. Conf. Comput. Appl. Syst. (COMPAS).

[10] Nofal M. (2023). A corpus-driven exploration of language use in religious discourse. J. Res. Appl. Linguist..

[11] M. Billah, et al. ``A systematic study and analysis of Bengali folklore with natural language processing systems.'' arXiv:2203.06607 (2022).

[12] Chatterji R. (2016). Scripting the folk: history, folklore, and the imagination of place in Bengal. Annu. Rev. Anthropol..

