# Supplementary material for: Dataset on multiregional variations of Bangla language (BD-Dialect)
Source: Data Brief. 2026 Mar 3;66:112654. doi: 10.1016/j.dib.2026.112654 (PMC12996979; doi:10.1016/j.dib.2026.112654)
Supplement: Supplementary file 2 [file mmc2.pdf]

BD-Dialect: Elicitation and Validation Protocol (v2.0)

1. PARTICIPANTS

Elicitation Phase: 3-5 native speakers per dialect (total 19). Born/raised in region, secondary education minimum, age 20-32.

Validation Phase: Three independent native speakers per dialect (different from elicitation speakers).

2. DATA COLLECTION

Structure: 950 parallel entries across 7 columns: Standard Bangla, English, and 5 dialect forms.

Method:

1. Word List: 200 common concepts (objects, actions, people, nature, abstract terms)
2. Clause List: 750 everyday sentences (greetings, commands, descriptions, questions)
3. Prompt: "How would you say this naturally in your local dialect with family/friends?"
4. Format: One-on-one sessions, recorded (audio for verification)

3. VALIDATION PROCESS

1. Independent Review: 3 validators per dialect rate each entry: Accept/Modify/Reject
2. Consensus Building: Entries with disagreements → group discussion → consensus decision
3. Finalization: Only consensus-validated entries included (>95% unanimous agreement)

4. DATA PROCESSING

- Transcription: Bangla Unicode script with phonetic romanization
- Files: Words.csv and Clauses.csv (950×7, UTF-8 encoding)
- Quality: Cross-checked with literary sources, audio verification for phonetics

5. ETHICS

- Consent: Verbal consent obtained ("Your contributions will be anonymized for public research...")
- Anonymity: All personal identifiers removed, audio files coded
- Access: Dataset publicly available via Mendeley Data (CC BY 4.0)

6. LIMITATIONS

- Focuses on 5 major dialect groups (not exhaustive)
- 3-5 primary speakers per dialect
- Limited audio corpus (pilot recordings only)

Contact: Anika Rahman | Dataset DOI: 10.17632/k769s4vk5z.2
